# Choosing the right partner in hormone-dependent gene regulation: Glucocorticoid and progesterone receptors crosstalk in breast cancer cells

**DOI:** 10.3389/fendo.2022.1037177

**Published:** 2022-11-04

**Authors:** Adali Pecci, María Florencia Ogara, Rosario T. Sanz, Guillermo Pablo Vicent

**Affiliations:** ^1^ Departamento de Química Biológica, Facultad de Ciencias Exactas y Naturales, Universidad de Buenos Aires, Ciudad Universitaria, Buenos Aires, Argentina; ^2^ Instituto de Fisiología, Biología Molecular y Neurociencias (IFIBYNE), Consejo Nacional de Investigaciones Científicas y Técnicas (CONICET)-Universidad de Buenos Aires, Ciudad Universitaria, Buenos Aires, Argentina; ^3^ Molecular Biology Institute of Barcelona, Consejo Superior de Investigaciones Científicas (IBMB-CSIC), Barcelona, Spain

**Keywords:** glucocorticoid receptor, progesterone receptor, mammary epithelial cell proliferation, chromatin, gene expression-regulation

## Abstract

Steroid hormone receptors (SHRs) belong to a large family of ligand-activated nuclear receptors that share certain characteristics and possess others that make them unique. It was thought for many years that the specificity of hormone response lay in the ligand. Although this may be true for pure agonists, the natural ligands as progesterone, corticosterone and cortisol present a broader effect by simultaneous activation of several SHRs. Moreover, SHRs share structural and functional characteristics that range from similarities between ligand-binding pockets to recognition of specific DNA sequences. These properties are clearly evident in progesterone (PR) and glucocorticoid receptors (GR); however, the biological responses triggered by each receptor in the presence of its ligand are different, and in some cases, even opposite. Thus, what confers the specificity of response to a given receptor is a long-standing topic of discussion that has not yet been unveiled. The levels of expression of each receptor, the differential interaction with coregulators, the chromatin accessibility as well as the DNA sequence of the target regions in the genome, are reliable sources of variability in hormone action that could explain the results obtained so far. Yet, to add further complexity to this scenario, it has been described that receptors can form heterocomplexes which can either compromise or potentiate the respective hormone-activated pathways with its possible impact on the pathological condition. In the present review, we summarized the state of the art of the functional cross-talk between PR and GR in breast cancer cells and we also discussed new paradigms of specificity in hormone action.

## Introduction

**Figure 1:**
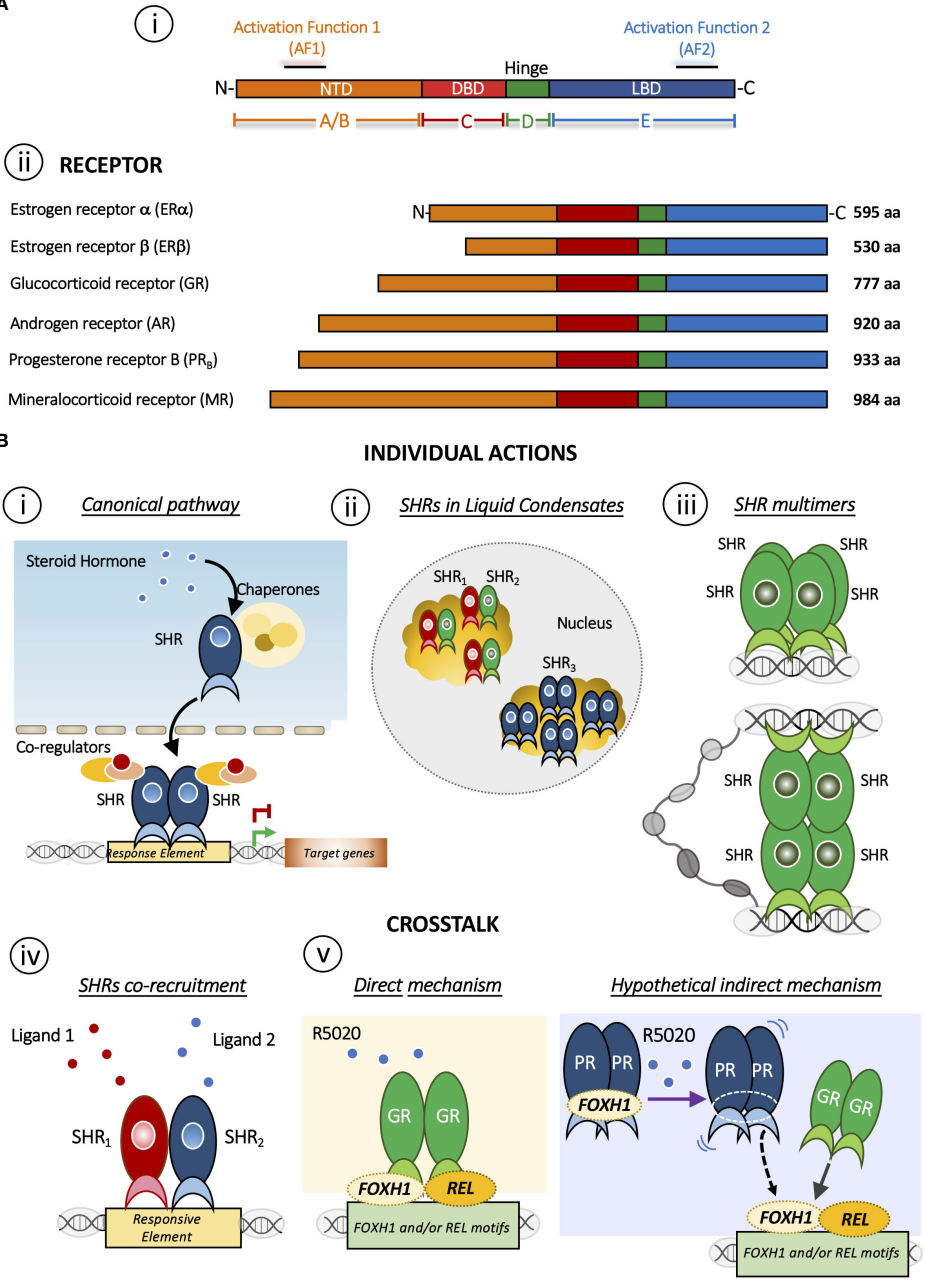
**(A)** Domain structure of SHRs. **i)** Basic domain structure of SHRs is composed of an unstructured N-terminal domain (NTD) that contains the Activation Function 1 (AF-1) surface, a zinc finger DNA-binding domain (DBD), a flexible hinge region, and a LBD that binds to ligands and interacts with co-regulator proteins through the Activation Function 2 (AF-2) surface. **ii)** Domain size and amino acid length of different members of the SHRs sub-family. The DBD and LBDs are the most conserved regions whereas the other domains are more variable in length and sequence composition. **(B)** Mechanisms of action of SHRs. Individual action **i)** The canonical pathway is shown. Steroid hormone binding to the steroid hormone receptor (SHR), often in the cytoplasm, causes the receptor to undergo a conformational change and translocate to the nucleus, where it interacts with specific DNA sequences to regulate transcription of target genes. **ii)** Distribution of SHRs in liquid condensates or foci in the nucleus [Reviewed in (4)]. **iii)** Steroid hormone receptor could form multimers or quaternary structure after DNA binding (5, 6). Interesting, this mechanism brings together regions that may be distant in the linear genome. Crosstalk between SHRs. **iv)** The SHRs co-recruitment to DNA response elements occurs in presence of both ligands. As a result, this crosstalk triggers a specific gene program. SHRs= ERα/PR (7); SHRs= GR/PR (8); SHRs= ERα/GR (9). **v)** Redirection mechanism in which the presence of R5050 in GC-free medium leads to the binding of GR to REL and FHOX1 motifs and repress the expression genes required for PR function (8). On one hand, it has been described that R5020 can directly activate and could drive GR binding (10) but R5020 activated PR could also participate indirectly stabilizing REL and/or FOXH1, increasing GR binding. This possibility, although feasible, requires further investigation.

**​​**Steroid hormones play diverse roles in the regulation of biological functions such as pregnancy, sex organ development, inflammation and immune responses, cholesterol distribution and brain function (1, 2). These effects are mediated by the members of the highly conserved Steroid Hormone receptor (SHR) sub-family that includes receptors for estrogens (ER), progestins (PR), androgens (AR), glucocorticoids (GR) and mineralocorticoids (MR) (3) (**Figure 1A i**). In fact, a general structural organization is common to all nuclear receptor family members, although the regulation of their quaternary structure may differ (5).

The SHR´s structure has been extensively discussed in excellent reviews (11–13). Briefly, SHRs are composed of three different domains: an N-terminal ligand-independent activation function 1 domain composed of an intrinsically disordered region (AF-1), a central DNA-binding domain (DBD), which links to the C-terminal ligand-binding and the activation function 2 domain (LBD/AF-2) through a hinge region. Particularly, the AF-2 domain is a primarily hydrophobic groove formed by residues from helices H3, H4 and H12 of the LBD/AF-2 domain, where the H12 position plays a critical role in the AF-2 spatial conformation and SHRs function. In fact, AF-2 interacts with specific residues present in particular coregulators’ amino acid motifs (LxxLL and I/LxxII for coactivators and corepressors families, respectively) arranged on one side of their amphipathic helix (7). Here we will focus on PR and GR, two receptors that shared several features ranging from several aspects of ligand-binding to the DNA sequences to which the receptor binds (**Figure 1A ii**). In the absence of ligands, these receptors are part of a protein complex associated with chaperones and co-chaperones, which increase the affinity of SHRs to their ligands *in vivo* (14). Early reconstitution experiments with GR (15) and with PR established that the central proteins in the activation pathway include Hsp40, Hsp70, Hsp90, HOP, and p23 (16, 17). Recently, has been shown that coordinated chaperone interactions enhance stability, function and regulation of GR (18).

Activation occurs when the ligand interacts with the receptor and initiates a signal transduction cascade which ultimately leads to changes in gene expression, whose canonical pathway is depicted in **Figure 1B i**. PR and GR present a heterogeneous distribution concentrated in liquid condensates or foci (**Figure 1B ii**). The formation of these discrete foci (19–29), containing ~40-80 receptor molecules have been reported (30). This nuclear compartmentalization would modulate the kinetics of biochemical reactions and thus would actively participate the transcription process (reviewed in (4)).

SHR´s mechanism of action involves genomic and nongenomic processes. Genomic actions result from the direct binding of ligand-activated receptor complexes to specific hormone responsive DNA elements located at the enhancers and promoters of target genes; and/or the ligand-receptor recruitment to other regions in the genome, relying on additional transcription factors such as FOXA1, GATA-3, STAT5, NFκ-B, and AP-1, among others (31, 32).

Several families of co-activators interact directly with ligand-receptor complexes. Some of them do so through the AF-1 domain (33, 34), while others by means of the LBD/AF-2 (35). In this way, the regulation of gene transcription by these receptors is closely associated with the reorganization of chromatin at target genes.

In addition to these direct genomic effects, steroid hormones induce rapid nongenomic responses similar to those initiated by peptide growth factors (36, 37). For example, progestins can activate Src/p21ras/Erk and the PI3K/Akt pathways, either *via* an interaction of the PR with ERα, which itself activates c-Src and PI3K, or by direct interaction of PR with the SH3 domain of c-Src (38–40). A growing body of evidence suggests that GR may also act *via* nongenomic mechanisms. Glucocorticoid activation of a membrane associated GR regulates gap junction intercellular communication and neural progenitor cell proliferation by a mechanism that requires c-Src activity and rapid MAPK-dependent phosphorylation of connexin-43 (41).

Traditionally, the genomic and nongenomic actions of steroid hormones have been considered as two independent pathways, but we found that both pathways converge in the modification of structural components of the target chromatin (42).

The breast develops predominantly after birth: a poorly developed ductal system initially begins to unfold during puberty and gains in complexity during adulthood (43). From pregnancy to lactation, lobuloalveolar growth is followed by the complete differentiation of the mammary epithelium and at weaning, a dramatic switch from survival to death signaling occurs, leading to mammary gland involution. During these periods experienced throughout a woman’s life, hormones promote first mammary gland development resulting in ductal elongation, then in adulthood, through recurrent estrous cycles trigger side branching and upon each pregnancy they control cyclical periods of cellular proliferation, differentiation and regression of the mammary epithelium (44). While ER is required at an earlier stage to induce ductal elongation, PR is needed later for side branching and alveologenesis (44–46). Progestins were described to be involved in driving cell proliferation, thus favoring breast cancer development but also to inhibit ER-dependent breast tumorigenesis (7, 47). Moreover, progestins also inhibit the production and secretion of milk and stimulate the proliferation of epithelial cells during late pregnancy (48). On the other hand, glucocorticoids (GCs) play a key role at puberty and pregnancy (49, 50); they promote lactation and the synthesis of milk proteins, maintaining the differentiation stage of the mammary epithelium (51–53). GR is expressed in all stages from normal to cancerous breast tissue (54, 55).

## PR in mammary epithelial cells: Different mechanisms engaged depending on the cell type and clinical status

PR is expressed from a single gene as two main isoforms, PR_A_ (94 kDa) and PR_B_ (116 kDa) (56, 57), both are transcribed from two distinct promoters and exhibit different transcriptional and biological activities as ligand-activated transcription factors. PR_B_ is a full-length receptor; PR_A_ is a truncated form of PR_B_ lacking the N-terminal 164 amino acids (56, 58). Both isoforms are usually co-expressed at similar levels in normal breast while a significant increase in PR_A_ or PR_B_ was detected in breast cancer that correlates with lesion progression, from the normal state to malignancy. In this regard, the PR_A_/PR_B_ ratio has been proposed as a prognostic and predictive factor for antiprogestin responsiveness in breast cancer (59). Some reports indicated that a high ratio of PR_A_/PR_B_ is associated with worse prognosis, and recurrence after tamoxifen (60, 61), yet other studies concluded that higher PR_A_/PR_B_ are related with biomarkers of better prognosis (59, 62). These contradictory results speak clearly that the mechanism of action of PR is more complex than we originally thought and requires further investigation. Moreover, PR_A_ and PR_B_ can form homodimers or heterodimers that exhibit distinct transcriptional regulatory functions by targeting different subsets of genes (63–66).

In the mammary gland, the luminal epithelium forms the inner layer of the ducts and the basal epithelium harbors myoepithelial cells that form the outer layer of the mature mammary ducts as well as stem and progenitor cells. The mammary ducts also comprise fibrous connective tissue, and variable amounts of adipose tissue (43, 67). In the adult mouse mammary gland, PR is expressed only in a fraction of the luminal epithelial cells, where progesterone promotes alveolar growth by a paracrine mechanism. In PR^+^ breast luminal cells progesterone upregulates RANKL expression. Then, RANKL binds to RANK expressed on the surface of neighboring PR^-^ luminal cells or basal cells, activating downstream pathways of cell proliferation, expansion, and survival. Progesterone can also induce adjacent PR^+^ cell proliferation by a cell-autonomous, CCND1-dependent mechanism. Moreover, progesterone also elicits the proliferation of PR^-^ luminal epithelial cells by a paracrine mechanism involving RANK and the NFκB signaling pathway (68).

Most of the research performed so far has been carried out in breast cancer cell lines, with very few studies conducted in normal human breast. Therefore, the role of PR in mammary physiology is underrepresented. This is justified by the loss of receptors detected in normal mammary cells once in culture. However, some steps have already been taken to overcome this issue. In 2009 Graham et al. reported the development and validation of a physiologically relevant model of matrix-embedded normal human breast cells, which was appropriate for studying hormone action in the normal breast (69). Moreover, Clarke et al. performed genome wide PR binding studies in breast cancer cells and in immortalized normal breast cells (70). Although PR binding was correlated with transcriptional outcome in both cell lines, there was a remarkably low overlap between the PR cistromes and in transcriptional targets. Moreover, distinct patterns of enrichment of known cofactor binding motifs were detected, with FOXA1 sites over-represented in breast cancer binding regions and NF1 and AP-1 motifs uniquely enriched in normal cells (70). What determines this difference? The expression levels and/or activation of the cofactors that participate in PR signaling could explain these variations. Hence, the pioneer factor FOXA1 would be a tumoral cofactor of PR. Conversely, NF1 and AP-1 would be the transcription factors chosen by PR in the normal context. An in-depth analysis of the differences in gene expression and PR binding in systems that adequately recapitulate both normal and breast cancer systems, may provide clinically valuable information on hormonal action. Thus, cofactor levels may modulate PR specificity (70).

Functional studies, performed in human breast, support that PR^+^/ER^+^ cells do not proliferate in direct response to hormone signals, but rather exert a paracrine effect on the surrounding PR^-^/ER^-^ cells (71, 72). In contrast, almost 70% of breast cancers express ER and PR and require their ligands during breast cancer progression, suggesting that these cells switch from paracrine to autocrine mechanisms, as they acquire the ability to proliferate during the tumorigenic process. However, the mechanisms that drive this “switch” are not known. In this regard, the presence of tumoral cofactors such as FOXA1 or the availability of other receptors could help to redirect PR and/or ER binding towards an oncogenic program. Indeed, as it has been described for ER and PR, the formation of receptor heterocomplexes in the presence of both ligands could regulate the process towards malignancy in PR^+^/ER^+^ cells (7).

## GR in mammary epithelial cells: Its dual role in cell proliferation/differentiation depending on the cellular context

It is well known that GR mediates the effects of stress hormones, and of synthetic derivatives that are widely used in the clinic as anti-inflammatory and immunosuppressive agents (73). In the mammary gland, GR was found strongly localized in the nuclei of myoepithelial cells surrounding lobular and duct units and occasionally localized in the nuclei of stromal and endothelial cells (74). The GR nuclear localization indicates that the receptor is transcriptionally active, since the inactive GR resides mainly in the cytoplasm bound to heat shock proteins and immunophilins (14, 75).

In mammary development, GR was shown to be essential for cell proliferation during lobulo-alveolar development and to contribute to mammary lobular unit spatial formation (50). Of note, GCs also exert anti-proliferative and anti-apoptotic activity in mammary epithelial breast cancer cell lines (76, 77). These steroids are used in the treatment of metastatic breast cancer to reduce the side effects produced by the chemotherapy, and to treat symptoms related to advanced cancer. However, despite the fact that GR expression in mammary tissue declines from normal to precancerous lesions and to invasive breast carcinoma (54, 78), the increment in stress hormones during breast cancer progression results in GR activation even at distant metastatic sites. This, in turn, increases intra-tumor heterogeneity and colonization, therefore reducing cell survival. These observations suggest that caution is needed when including GCs in the treatment of breast cancer patients (79).

Activated GR undergoes phosphorylation, oligomerization and nuclear translocation (80). In the nucleus, the receptor is predominantly recruited to pre-accessible sites along with chromatin remodeling enzymes (81). Interestingly, GR is also able to initiate DNAse hypersensitive sites as a pioneer factor (82–84).

Like PR, chromatin remodeling factors regulate GR binding to DNA and thus, are involved in the overall function of GR. This points out the chromatin landscape as a major contributing factor to the GR-regulated cell-type specific gene expression (84–90). In fact, Johnson et al. reported that all GR binding events involving the SWI/SNF remodeling complex are either pre-recruited by other factors or recruited by the receptor itself (88).

In mouse mammary epithelial cells, 51% of pre-programmed GR binding sites are enriched in the pioneer transcription factor AP-1 (89), which along with GR triggers the recruitment of several remodelers such as Brg1, Chd4, and Snf2h (91). Nevertheless, activated GR induces *de novo* remodeling of chromatin at a minority (~15%) of GR binding sites in a highly tissue-specific manner (85, 89, 90, 92, 93). The current evidence suggests that in mammary epithelial cells this pioneering capacity of AP-1 would be relevant for regulating chromatin accessibility not only at GR target enhancers but also at other genomic regions (89).

The oligomeric status of the GR has also been considered to play a key role in the mechanism of action of the activated receptor. Moreover, in the late 1990s the oligomerization state was proposed as a parameter in the search for synthetic ligands with dissociate GC effects (6). In this sense, we have reported that hormone-activated GR adopts a dimer configuration in the nucleus of living murine mammary adenocarcinoma cells (94) and upon binding to a specific DNA binding site the GR dimer becomes a tetramer (**Figure 1B iii**) (5, 6). Of note, tetrameric configuration was also detected in activated PR complexes in the same cells (5). The influence of DNA binding on the quaternary structure of the GR proposes a similarity to an allosteric structural transition of the receptor once bound to its target DNA region (95–97).

## Functional crosstalk between SHRs: Complexity comes to the forefront

Although at different concentrations, various hormones are present simultaneously in the bloodstream or locally at their target cells. For instance, estrogens that are governed by the menstrual cycle coincide at any time with high levels of GCs, which are regulated by stress and circadian cycles. These multiple signals converge to the same cell and, together, they participate in the cellular response. In this sense, the mechanisms of hormonal action need to be studied in an integrated manner, where different receptors could be activated simultaneously by their cognate ligands.

GCs exert an antagonistic effect on estrogen-dependent cell growth in ER^+^/GR^+^ breast carcinoma cells and reduce their cell proliferation through a functional crosstalk between both receptors (**Figure 1B iv**) (9, 32, 98–100). On the other hand, also in breast cancer models, it has been proposed that PR redirects ERα chromatin binding events. ERα and PR form a complex in the presence of both ligands, resulting in a unique gene expression program that is associated with good clinical outcome (**Figure 1B iv**) (7). In this case, it has been proposed that PR functions as a molecular rheostat to control ERα chromatin binding and transcriptional activity.

Furthermore, it was recently reported the generation of metastasis-competent circulating tumor cells (CTCs) in patients with breast cancer occur during sleep, in the rest phase; while CTCs generated during the active phase are devoid of metastatic ability. The authors found that key circadian rhythm hormones such as melatonin, testosterone and GCs dictate CTC generation dynamics (101). Treatment with the synthetic GC dexamethasone (DEX) or testosterone did not affect primary tumor size but resulted in a marked reduction in single CTCs and CTC clusters (101). These key effects of hormones that determine the metastatic capacity of cancer cells are likely to occur in the presence of both GCs and androgens, thus, a putative crosstalk between both activated receptors could be directly operating in this process.

Unlike the functional connections between ERα with PR and GR, or AR with ERα and GR, few studies have addressed the influence of GR on the transcriptional activity of PR and *vice versa*. Similarities in protein structure as well as in the DNA sequences to which the receptors bind are readily evident for PR and GR (>90% sequence identity between their DNA binding domains (DBD). However, the resulting biological response differs markedly. For example, the association of progestins with breast cancer incidence and progression contrasts with the growth suppressive action of GCs on ER^+^/PR^+^ breast tumor cells (102, 103). In fact, in those cell types, increased circulating levels of progestins and estrogens and/or overexpression of their receptors lead to uncontrolled cell division (47, 104). On the other hand, previous works suggested that GR would act as a suppressor of proliferation (52, 105) as well as a cell death inducer in tumoral mammary epithelium (106).

To address the existence of a potential crosstalk between PR and GR, we used PR^+^/GR^+^ breast cancer cell lines (107, 108) where we found that GC-free or DEX-activated GR inhibits PR-dependent cell proliferation and dedifferentiation through the modulation of certain PR-target genes, i.e. *GREB1, STAT5A, ELF5* and *SNAI1* (8). In the presence of DEX, the antagonistic effect increases and involves the formation of GR-PR protein complexes. By ChIP-seq and sequential ChIP analyses, we detected overlapped binding of GR and PR at key enhancer sites and confirmed co-recruitment of both receptors to shared sites (**Figure 1B iv**). Moreover, GC-free GR upon stimulation with the PR-agonist R5020, can bind to REL and FOXH1 motifs and repress the expression of nearby genes encoding for SWI/SNF and other chromatin remodeler complexes such as SMARCD2, ARID1A and INO80C (**Figure 1B v**). Thus, in the presence of the synthetic progestin R5020, relocated GR are bound to a subset of genes required for PR function, reinforcing the anti-progestational effect of GCs in ER^+^/PR^+^ breast cancer cells (8).

The mechanism behind the R5020-dependent GR binding to approximately 600 unique sites could be due at least by two non-mutually exclusive mechanisms: 1) a direct effect of R5020 on the GR or 2) an indirect mechanism whereby activated PR would stabilize GR binding to other transcription factors (i.e. REL or FOXH1) genomic regions (**Figure 1B v**).

Regarding the direct effect, although R5020 is considered a PR specific agonist, it has been reported that R5020 can also activate GR (10). Thus, R5020 binding to GR would induce a unique conformational change to the receptor leading to its recruitment to REL and/or FOXH1-enriched regions. However, we can also speculate that R5020-activated PR could favor GR recruitment to REL and/or FOXH1 regions throughout an indirect mechanism. Accordingly, it has been reported that FOXH1 can act as a hormone-independent corepressor of AR in prostate cancer cells; indeed, a protein-protein interaction was identified between the AR AF-1 domain and FOXH1 independently of the presence of dihydrotestosterone (109). In this model, R5020-activated PR could release FOXH1/REL, which in turn would bind to their sites in the genome favoring GR loading (**Figure 1B v**, right panel). Whether GR and PR physically interact with FOXH1 and/or REL in mammary cells is unknown and more research is required to support this second hypothetical mechanism.

## Conclusions and perspectives

Given the concentration of different steroid hormones varies considerably over a wide range of time (from hours, days and even weeks), several receptors might be coexisting and simultaneously activated by their ligands. Thus, the mechanism of control of one receptor over the other/s could be very frequent and could be involved in relevant receptor-mediated functions. This brings up a complex scenario in which several activated hormone receptors could be interacting reciprocally, regulating the final transcriptional output and their function in the target cell. This crosstalk between receptors could be positive, implying synergism between hormone pathways, or on the contrary, negative, through competition for pathways, for co-regulators and for binding sites in the genome.

Thus, it will be key to elucidate the causes that establish this hierarchy between receptors. What determines which receptor leads and regulates the activity over the other/s? The circulating levels of ligands, receptor levels, and the cellular identity of the target cell (more adapted to respond to one stimulus than to another) are factors that could be involved in this complex scenario. Also, under these circumstances, heterologous complexes, composed of different activated receptors and with different stoichiometry, could be formed and reciprocally regulate the hormonal pathways involved.

We propose that the prevalence of one or the other mechanism is dependent upon which ligand and/or combination of ligands bind to each receptor. Under this hypothesis, new questions arise regarding GR and PR functional crosstalk. How does the ligand-dependent conformation acquired by each receptor influence the control of gene expression? Do the GR-PR heterocomplexes display the same activity as homo-PR or homo-GR complexes? Do the GR-PR heterocomplexes recruit the same set of co-regulators? Do they have similar intranuclear dynamics compared to homodimers? Recruitment of the GR to non-canonical sites requires the presence of the transcription factor that directly recognizes that site, or does it do so by a different mechanism?

To address these questions, state-of-the-art techniques that allow us to monitor simultaneously homo- and heterocomplexes populations of PR and GR in the same cell, are required. For this purpose, it is necessary to engineered cell lines through mutations targeting the interacting regions involved in the formation of homo- and heterodimers. Moreover, mass spectrometry will enable to identify the protein interactome of each receptor population; Next Generation Sequencing (NGS) including ChIP-seq and RNA-seq will help to decipher the defined cistromes for each receptor population; and high-resolution microscopy will allow to visualize the nuclear dynamics of homo- or hetero-complexes in response to hormone, including the formation of condensates. This will shed light on the complex mechanism by which SHRs act upon simultaneous activation. Therefore, and from a pharmacological point of view, understanding PR-GR crosstalk could contribute to the design of new endocrine combined therapies that minimize tumor resistance, colonization, and metastasis and thus, provide tumorigenesis vulnerability for therapeutic improvement in patients.

## Author contributions

GV and AP wrote the manuscript. MO and RS searched for references and built the Figure. All authors contributed to the article and approved the submitted version.

## Funding

Grants from the Agencia Estatal de Investigación AEI/10.13039/501100011033 [PID2019-105173RBI00]; Agencia Nacional de Programación Científica y Tecnológica, [Préstamo BID-PICT2019-0158] and University of Buenos Aires [20820180100745BA], Argentina. MO and AP are members of the CONICET-Argentina. GV is Researcher of the Spanish National Research Council (CSIC), Spain.

## Acknowledgments

Authors thanks Dr. Diego M. Presman (IFIBYNE, University of Buenos Aires-CONICET) for advice on the manuscript.

## Conflict of interest

The authors declare that the research was conducted in the absence of any commercial or financial relationships that could be construed as a potential conflict of interest.

## Publisher’s note

All claims expressed in this article are solely those of the authors and do not necessarily represent those of their affiliated organizations, or those of the publisher, the editors and the reviewers. Any product that may be evaluated in this article, or claim that may be made by its manufacturer, is not guaranteed or endorsed by the publisher.
